# A Case of Three-Vessel Coronary Artery Disease Associated With Marijuana Use

**DOI:** 10.7759/cureus.14481

**Published:** 2021-04-14

**Authors:** William Lim, Maham Suhail, Sean Galligan

**Affiliations:** 1 Internal Medicine, Richmond University Medical Center, Staten Island, USA; 2 Internal Medicine/Cardiology, Richmond University Medical Center, Staten Island, USA

**Keywords:** multivessel coronary artery disease (mvcad), marijuana use, primary percutaneous coronary intervention (pci), recreational drug use

## Abstract

Marijuana is one of the most popular psychotropic drugs among adolescents and young adults. With the recent surge in marijuana use across the United States, it is very important for physicians to understand the clinical implications associated with marijuana use. In this case report, we discuss a case of a young adult who presented to the emergency department with chest pain and was found to have three-vessel coronary artery disease (CAD). The patient did not have any significant past medical history or family history of cardiac diseases but reported a significant history of marijuana use. This case report aims to add to the growing area of research on the association between myocardial infarction (MI) and marijuana use.

## Introduction

Myocardial infarction (MI) is most commonly caused by atherosclerosis and/or inflammatory processes of coronary artery walls. However, MI can also be caused by drugs, chemotherapeutic agents, and psychotropic drugs [1]. Marijuana (cannabis) is a psychotropic drug known to have several well-described effects on the cardiovascular system, such as tachycardia, arrhythmia, hypertension, and orthostatic hypotension [2]. Although pathophysiologic mechanisms of marijuana-induced MI are unclear, there have been documented cases of MI associated with marijuana use. In this report, we present a case of a 32-year-old male with no significant past medical history who presented to the emergency department (ED) with chest pain and was found to have three-vessel coronary artery disease (CAD). The patient had a significant history of marijuana use.

## Case presentation

**Figure 1:**
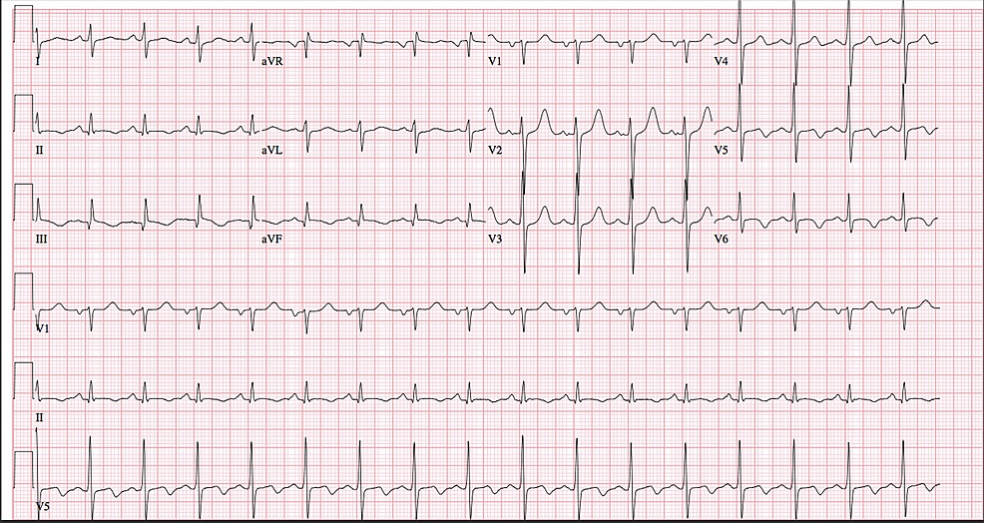
Electrocardiogram showing T-wave inversion in lateral leads (V5 and V6) and inferior leads (II, III, and aVF)

A 32-year-old man with no significant past medical history presented with a three-week history of intermittent, midsternal, non-radiating burning chest pain, which was worsened by strenuous activity and relieved by rest. He stated that the current episode of chest pain had started at midnight prior to the day of presentation and was associated with jaw pain and nausea. He denied any smoking history or family history of CAD or lipid disorder. His social history was significant for a 12-year history of marijuana use with his last use in the immediate past week. At presentation, his vitals were as follows: temperature of 97.9 °F, blood pressure of 177/138 mmHg, pulse rate of 106 bpm, and oxygen saturation of 98% on room air. His body mass index (BMI) was found to be 30 kg/m^2^. The patient's physical examination was normal. Chest radiography revealed no abnormalities. Troponin level on admission was 32.8, and the urine drug screen was positive for marijuana; his hemoglobin A1c (HbA1c) level was 5.4%. The initial electrocardiogram (EKG) upon presentation (Figure 1) was significant for T-wave inversions in lateral leads (V5 and V6) and inferior leads (II, III, and aVF).

**Figure 2:**
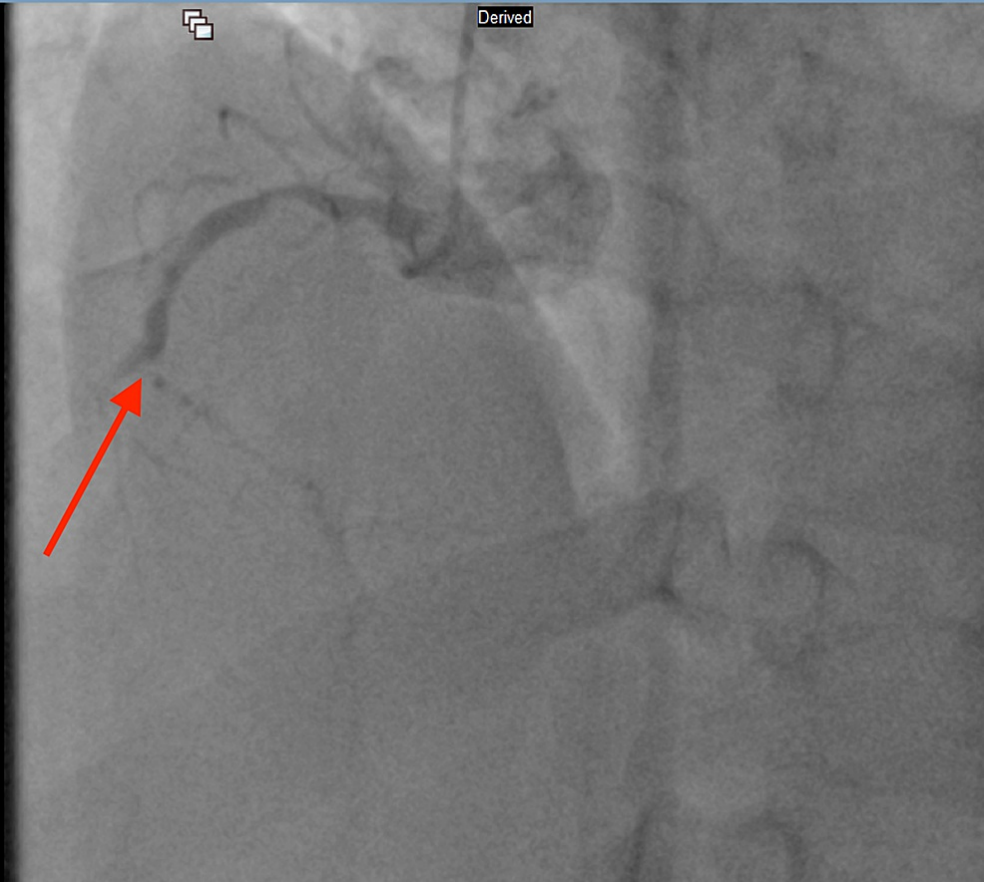
Right coronary artery occlusion from left anterior oblique cranial view (red arrow)

**Figure 3:**
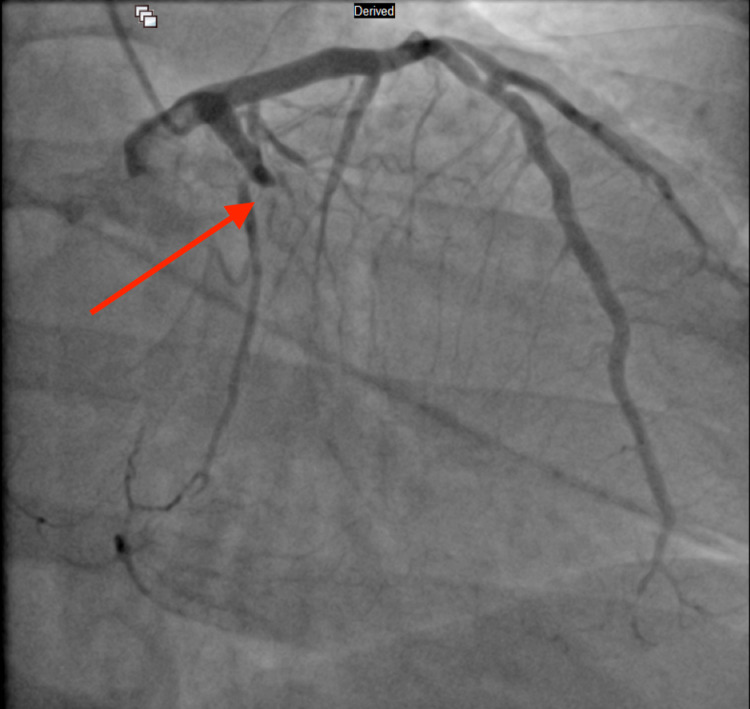
Left circumflex coronary artery occlusion from right anterior oblique caudal view (red arrow)

**Figure 4:**
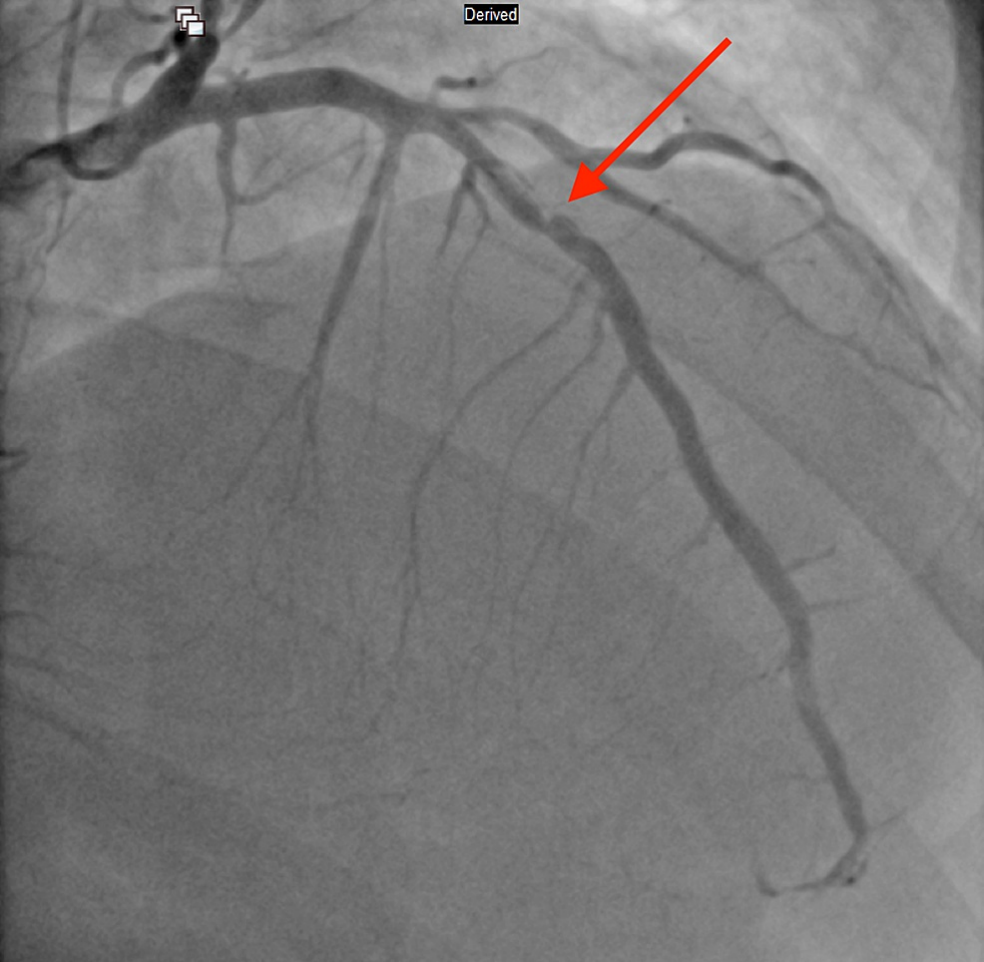
Middle left anterior descending artery occlusion from right anterior oblique cranial view (red arrow)

**Figure 5:**
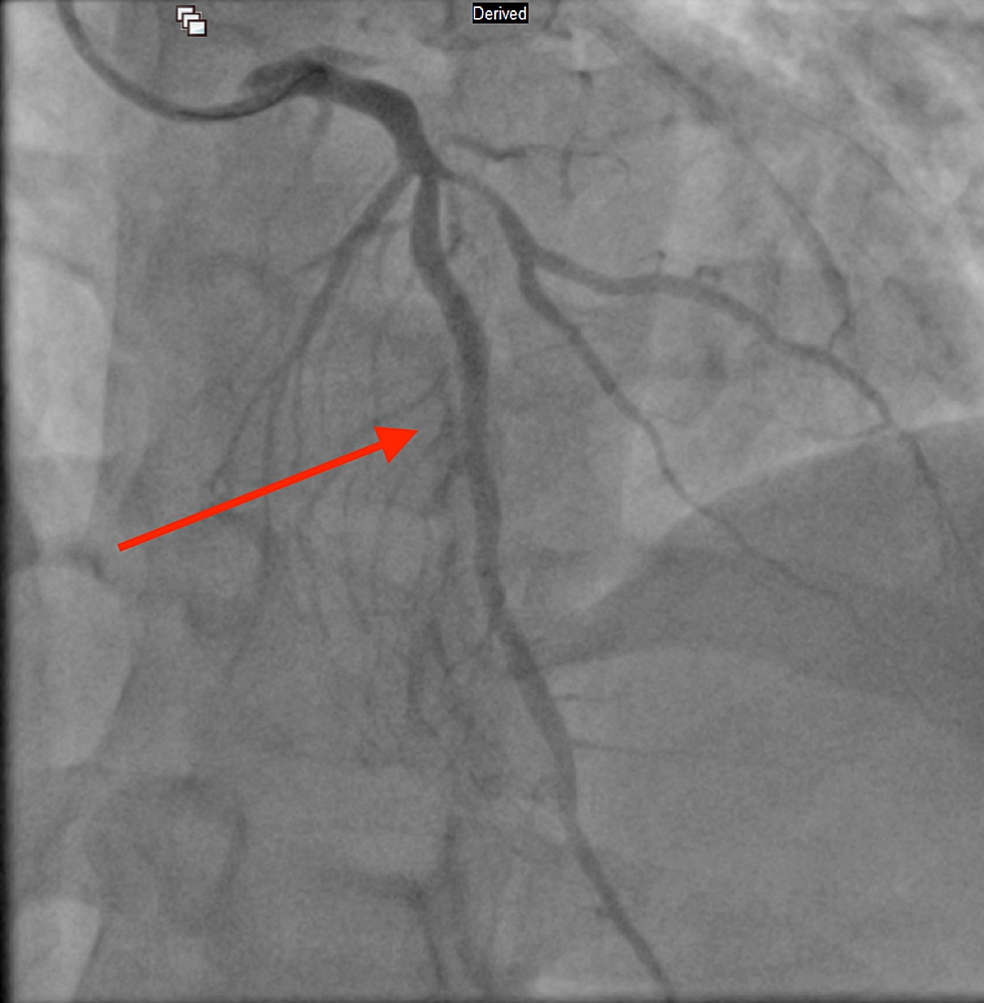
Post-percutaneous coronary intervention of middle left anterior descending artery from left anterior oblique cranial view (red arrow)

**Figure 6:**
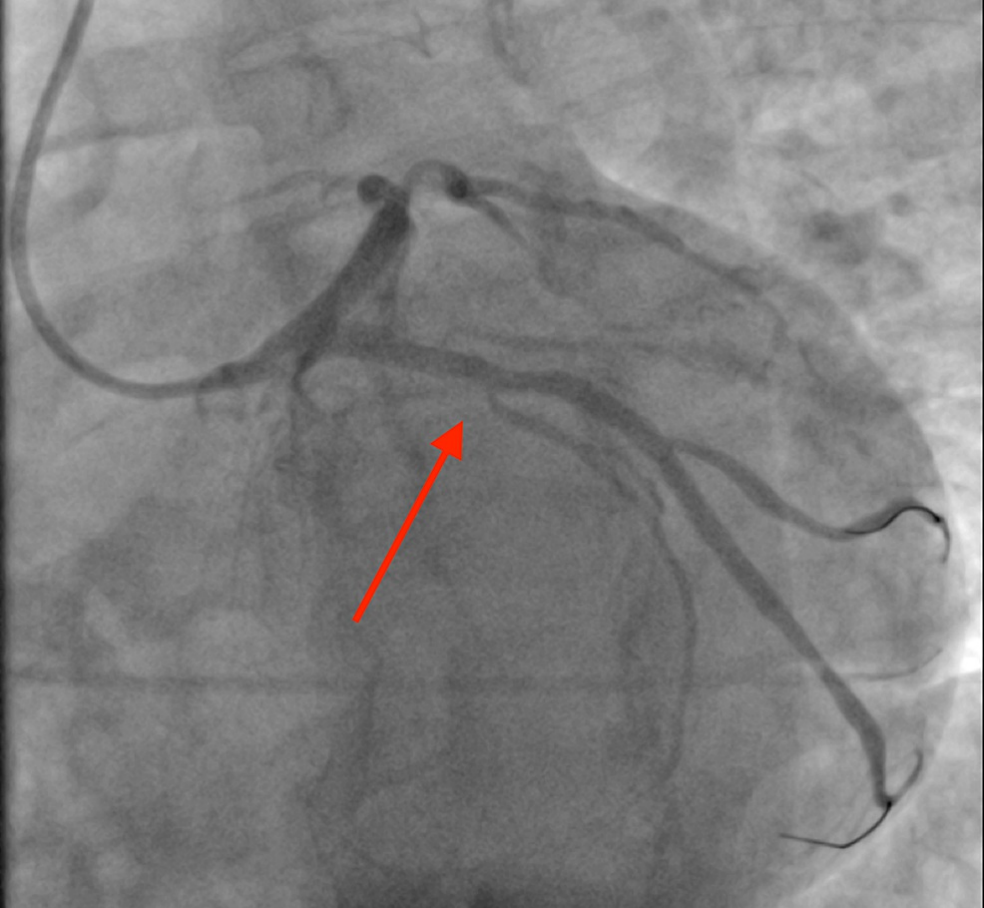
Post-percutaneous coronary intervention of left circumflex coronary artery from left anterior oblique caudal view (red arrow)

Due to the excessively high troponin level, an emergency cardiac catheterization was performed, which revealed three-vessel CAD [100% occlusion of the right coronary artery (RCA) and obtuse marginal artery (OM1), 90% occlusion of left anterior descending artery (LAD) and 60% stenosis of distal left circumflex artery (LCx)], and a drug-eluting stent was placed in the middle LAD and OM1. RCA occlusion from left anterior oblique (LAO) cranial view, LCx occlusion from right anterior oblique (RAO) caudal view, and middle left anterior descending artery occlusion from RAO cranial view can be seen in Figure 2, Figure 3, and Figure 4 respectively. Post-percutaneous coronary intervention (PCI) of middle LAD from LAO cranial view and LCx from LAO caudal view can be seen in Figure 5 and Figure 6 respectively.

The lipid panel of the patient showed triglycerides of 800 mg/dl, total cholesterol of 230 mg/dl, and high-density lipoprotein (HDL) of 22 mg/dl. Low-density lipoprotein (LDL) and very-low-density lipoprotein (VLDL) were unable to be calculated because of high triglyceride levels (>400 mg/dl). An echocardiogram after the cardiac catheterization showed moderate left ventricular systolic dysfunction and an estimated left ventricular ejection fraction of 40%. The patient remained well after the procedure and was discharged home 48 hours later.

The patient was counseled about the importance of quitting marijuana use, and he was discharged home with amlodipine (5 mg oral daily), atorvastatin (80 mg oral at bedtime), hydrochlorothiazide (25 mg oral daily), losartan (50 mg oral daily), metoprolol succinate (50 mg oral daily), aspirin (81 mg oral daily), and ticagrelor (90 mg oral twice a day).

## Discussion

Marijuana is the second most commonly abused psychotropic drug in the United States, after alcohol. Approximately 147 million people worldwide and 24 million people in the United States have reported using marijuana [3], and more than two million adults in the United States with established cardiovascular diseases were found to be currently using marijuana or have used marijuana in the past [4].

Marijuana smoking is known to have a modest, short-lived increase in the risk of acute MI, even in individuals without any associated cardiac risk factors. A prospective study involving 3,882 patients with acute MI showed that 3.2% of them had reported smoking marijuana in the past year. This study also reported that the risk of the onset of MI is elevated 4.8 times in the first hour after marijuana use [5]. Weekly cannabis users were found to have 4.2 times increased risk of mortality compared to non-users in a study involving 1,913 patients with acute MI [6]. 

The pathogenesis of cannabis-induced MI may be multifactorial and includes increased myocardial oxygen demand, decreased blood supply to the myocardium, marked vasoconstriction of the coronary arteries, and platelet activation. Marijuana can acutely increase sympathetic activity and decrease parasympathetic activity, leading to tachycardia, vasodilation, and an increase in cardiac output and myocardial oxygen demand with little or no increase in blood pressure. These acute changes probably account for the association between cannabis smoking and acute MI [7,8].

The activation of cannabinoid receptor type 1 (CB1) by cannabis has been shown to be pro-atherogenic due to increased reactive oxygen species (ROS), thereby promoting endothelial injury and increasing lipid accumulation in macrophages [9]. In the Strategy To Reduce Atherosclerosis Development InVolving Administration of Rimonabant - the Intravascular Ultrasound Study (STRADIVARIUS) trial, rimonabant (CB1 receptor antagonist) was shown to decrease the total atheroma volume. Sugamura et al. have also found that rimonabant can reduce atherosclerosis in the animal model [10]. These studies show that there is an extrapolation to suggest the increased risk of atherogenesis with CB1 agonism, though further studies are needed to establish this [5]. The patient's significant history of marijuana use could explain his hyperlipidemia, especially since he was young in age with no significant family history of CAD or lipid disorders, which could, in turn, lead to CAD.

Another theory suggests that marijuana can increase the expression of glycoprotein IIb-IIIa and P-selectin by activating the cannabinoid receptors in the platelet. These proteins are responsible for the final pathway of platelet aggregation and they likely create a prothrombotic state in otherwise healthy cannabis users [11]. Furthermore, smoking marijuana is associated with an increase in carboxyhemoglobin, leading to decreased oxygen transport to the heart [12].

In our case, a young patient with no significant cardiac risk factors who reported the use of marijuana for 12 years was found to have three-vessel CAD. His significant marijuana use could directly have led to CAD or could have contributed to hyperlipidemia, which in turn may have led to CAD. We can extrapolate from this case that there is some association between marijuana use and CAD although further studies are needed to establish this connection.

## Conclusions

This case illustrates the association between MI and marijuana use and adds to the growing body of literature about the topic. It also highlights the importance of proper history taking, especially drug-related history in young patients presenting with chest pain. Since 11 states in the United States have already legalized the recreational use of marijuana and even more states are set to join this club in the near future, we need to raise awareness about marijuana use's effect on the cardiovascular system and perform further studies on the association between marijuana use and MI.

## References

[1] Kameczura T, Bryniarski L, Surowiec S, Kocowska M, Kawecka-Jaszcz K, Czarnecka D (2013). Myocardial infarction caused by pharmacological substances - case description and literature review. Postepy Kardiol Interwencyjnej.

[2] Goyal H, Awad HH, Ghali JK (2017). Role of cannabis in cardiovascular disorders. J Thorac Dis.

[3] (2021). Marijuana statistics 2020/2021. https://comparecamp.com/marijuana-statistics.

[4] DeFilippis EM, Bajaj NS, Singh A (2020). Marijuana use in patients with cardiovascular disease: JACC review topic of the week. J Am Coll Cardiol.

[5] Mittleman MA, Lewis RA, Maclure M, Sherwood JB, Muller JE (2001). Triggering myocardial infarction by marijuana. Circulation.

[6] Mukamal KJ, Maclure M, Muller JE, Mittleman MA (2008). An exploratory prospective study of marijuana use and mortality following acute myocardial infarction. Am Heart J.

[7] Thomas G, Kloner RA, Rezkalla S (2014). Adverse cardiovascular, cerebrovascular, and peripheral vascular effects of marijuana inhalation: what cardiologists need to know. Am J Cardiol.

[8] Pacher P, Steffens S, Haskó G, Schindler TH, Kunos G (2018). Cardiovascular effects of marijuana and synthetic cannabinoids: the good, the bad, and the ugly. Nat Rev Cardiol.

[9] (2021). Marijuana and coronary heart disease. https://www.acc.org/latest-in-cardiology/articles/2016/09/22/08/58/marijuana-and-coronary-heart-disease.

[10] Sugamura K, Sugiyama S, Nozaki T (2009). Activated endocannabinoid system in coronary artery disease and antiinflammatory effects of cannabinoid 1 receptor blockade on macrophages. Circulation.

[11] Deusch E, Kress HG, Kraft B, Kozek-Langenecker SA (2004). The procoagulatory effects of delta-9-tetrahydrocannabinol in human platelets. Anesth Analg.

[12] Hollister LE (1986). Health aspects of cannabis. Pharmacol Rev.

